# The spatial extent of tauopathy on [^18^F]MK-6240 tau PET shows stronger association with cognitive performances than the standard uptake value ratio in Alzheimer’s disease

**DOI:** 10.1007/s00259-024-06603-2

**Published:** 2024-01-17

**Authors:** Thomas Gérard, Lise Colmant, Vincent Malotaux, Yasmine Salman, Lara Huyghe, Lisa Quenon, Laurence Dricot, Adrian Ivanoiu, Renaud Lhommel, Bernard Hanseeuw

**Affiliations:** 1 Nuclear Medicine Department, Cliniques Universitaires Saint Luc, Brussels, Belgium; 2 Institute of Neurosciences, Université Catholique de Louvain, Brussels, Belgium; 3 Neurology Department, Cliniques Universitaires Saint Luc, Brussels, Belgium; 4 WELBIO Department, WEL Research Institute, Avenue Pasteur, 6, 1300 Wavre, Belgium; 5 Department of Radiology, Gordon Center for Medical Imaging, Massachusetts General Hospital, Harvard Medical School, Boston, MA USA

**Keywords:** Tauopathy, F18-MK6240, Tau PET, SUVr, Biomarker of Alzheimer’s disease, Brain imaging

## Abstract

**Purpose:**

[^18^F]MK-6240, a second-generation tau PET tracer, is increasingly used for the detection and the quantification of in vivo cerebral tauopathy in Alzheimer’s disease (AD). Given that neurological symptoms are better explained by the topography rather than by the nature of brain lesions, our study aimed to evaluate whether cognitive impairment would be more closely associated with the spatial extent than with the intensity of tau-PET signal, as measured by the standard uptake value ratio (SUVr).

**Methods:**

[^18^F]MK6240 tau-PET data from 82 participants in the AD spectrum were quantified in three different brain regions (*Braak* ≤ *2*, *Braak* ≤ *4*, and *Braak* ≤ *6*) using SUVr and the extent of tauopathy (EOT, percentage of voxels with SUVr ≥ 1.3). PET data were first compared between diagnostic categories, and ROC curves were computed to evaluate sensitivity and specificity. PET data were then correlated to cognitive performances and cerebrospinal fluid (CSF) tau values.

**Results:**

The EOT in the *Braak* ≤ *2* region provided the highest diagnostic accuracies, distinguishing between amyloid-negative and positive clinically unimpaired individuals (threshold = 9%, sensitivity = 79%, specificity = 82%) as well as between prodromal AD and preclinical AD (threshold = 38%, sensitivity = 81%, specificity = 93%). The EOT better correlated with cognition than SUVr (∆R^2^ + 0.08–0.09) with the best correlation observed for EOT in the *Braak* ≤ *4* region (*R*^2^ = 0.64). Cognitive performances were more closely associated with PET metrics than with CSF values.

**Conclusions:**

Quantifying [^18^F]MK-6240 tau PET in terms of EOT rather than SUVr significantly increases the correlation with cognitive performances. Quantification in the mesiotemporal lobe is the most useful to diagnose preclinical AD or prodromal AD.

**Supplementary Information:**

The online version contains supplementary material available at 10.1007/s00259-024-06603-2.

## Introduction

In the last two decades, the development of positron emission tomography (PET) biomarkers in Alzheimer’s disease (AD) has been a breakthrough. PET offers a non-invasive tool for the in vivo observation of cerebral amyloid plaques and tau neurofibrillary tangles, the neuropathological hallmarks of AD. Recently, research criteria for AD have been proposed, incorporating amyloid, tau, and neurodegeneration (A/T/N) biomarkers [1]. Several studies have highlighted the leading role of amyloid and tau combination in predicting cognitive decline [2–4]. Amyloid PET is now approved for clinical use in both Europe and the USA, as its impact on clinical management has been demonstrated [5]. However, regional amyloid PET signal has not shown strong correlations with concurrent cognitive performances [6, 7]. In contrast, tau PET imaging is a promising technique for explaining cognitive deficits, as pathological studies have demonstrated that cognition better correlates with tau than with amyloid pathology [8]. Previous PET studies have shown that tau-PET signal intensity is closely associated with the severity of cognitive impairment [9] and has greater accuracy than amyloid PET or MRI in diagnosing preclinical or prodromal AD [10].

Most previous tau-PET studies have used signal intensity, such as the standard uptake value ratio (SUVr), to quantify tau pathology. However, since Paul Broca described his famous aphasia case in 1861, neurologists know that clinical symptoms depend on the anatomy of the lesion in the central nervous system, and not on the nature of the lesion [11]. Hence, in this study, our objective was to compare the diagnostic accuracies and the correlation with cognitive performance of tau-PET images providing the anatomical distribution of tau tangles. By measuring both SUVr and the spatial extent of tau deposits in predefined brain regions, we aimed to assess whether clinical impairment is more closely associated with the intensity or the extension of tau pathology in the brain. We used a second-generation tau PET tracer, [^18^F]MK-6240, enabling semi-quantitative measures in various neocortical regions and, notably, in the mesiotemporal lobe, distinguishing it from other tau PET tracers [12].

Inspired by oncological metrics commonly used in nuclear medicine, such as the metabolic tumor volume (MTV) and the total lesion glycolysis (TLG = MTV * SUVmean), we developed a method of analysis aiming to distinguish the spatial extent from the intensity information contained in the SUVr measure. Our hypothesis was that the extent metric would exhibit a closer association with cognition than the intensity metrics across all stages of the disease. Furthermore, we hypothesized that the extent within the medial temporal lobe would be more accurate in distinguishing early, preclinical AD stages, whereas the extent in the neocortex would be more accurate in diagnosing AD dementia.

## Methods

### Participants

The study included a total of 82 adults (aged 55–98), comprising 59 patients and 23 volunteers (Table 1). Patients were recruited through the Memory Clinic of the Cliniques Universitaires Saint Luc in Brussels, Belgium. Volunteers were enlisted for other clinical studies through mailbox announcements and advertisements in the hospital’s vicinity. We selected volunteers from this pool to form a group enriched in apolipoprotein (Apo) E4 carriers, matching the frequency of ApoE4 carriage observed in patients. Recruitment and examinations were conducted between June 2019 and February 2023. Exclusion criteria were non-AD neurodegenerative pathologies, focal brain lesions, major depression or psychiatric diseases, and alcohol or drug abuse. Informed consent was obtained from all individual participants included in the study, adhering to the principles of the Declaration of Helsinki. Ethical approval for the study was granted by the Ethics Committee of the Catholic University of Louvain (Date: 13/05/2019; Eudra-CT number: 2018-0034/73-94).

**Table 1:** Demographics

		CNamy −	preAD	proAD	ADdem	All
General	Number included	17	14	32	19	82
	Age (years) (SD; min–max)	65.7 (8.9; 56–85)	74.5 (8.6; 65–98)**	73.3 (6.5; 58–86)**	70.0 (8.4; 55–82)	71.2 (8.4; 55–98)*
	ApoE ε4 carriers (%)	63	58	68	62	63
	APOE missing data	1	2	10	6	19
	Gender. (% female)	53	36	53	47	49
	Education (years) (SD)	16.0 (0.0)	15.7 (1.1)	13.6 (3.7)*	14.3 (3.3)*	14.6 (3.0)*
Cognition	MMSE (SD)	29.0 (0.9)	28.4 (1.2)	26.1 (1.5)****	20.2 (2.8)****	25.7 (3.7)****
	Global cognitive Z-score (SD)	0.31 (0.28)	− 0.15 (0.42)**	− 1.54 (0.71)****	− 2.87 (0.68)****	− 1.26 (1.30)****
Imaging	MK-6240 SUV cerebellum (SD)	0.72 (0.19)	0.71 (0.12)	0.66 (0.15)	0.71 (0.17)	0.69 (0.16)
	Flutemetamol Centiloid value	8.8 (6.1)	46.1 (16.7)****	64.01 (24.9)****	101.0 (34.1)***	39.0 (36.3)**
	Centiloid missing data	2	6	27	14	49
CSF	P-tau (SD) (cut-off: 61 pg/ml)	32.0 (2.9)	64.5 (64.1)	85.4 (39.9)*	114.8 (64.1)*	90.8 (52.6)
	T-tau (SD)) (cut-off: 381 pg/ml)	256.5 (48.8)	421.2 (317.6)	551.7 (242.1)*	726.3 (411.4)*	583.8 (327.6)
	AB42 (SD)) (cut-off: 437 pg/ml)	799.5 (95.5)	360.7 (70.6)	388.9 (126.8)**	398.3 (154.9)*	403.8 (150.5)**
	CSF missing data	15	8	3	1	27

Demographics table compiling general, cognition, PET, and CSF data. Differences between CNamy − and other groups were tested with Mann–Whitney test, and < 0.05 *p*-values are displayed as asterisk (*p < 0.05; **p < 0.01; ***p < 0.001; ****p < 0.0001)

*CNamy − *cognitively normal amyloid-negative subjects, *preAD* cognitively normal amyloid-positive subjects, *proAD* mild cognitively impaired amyloid-positive subjects, *ADdem* amyloid-positive demented subjects

All participants, patients and volunteers were pooled and given an exclusive categorical diagnosis. The diagnosis was based on two criteria: the clinical diagnosis, derived from their cognitive performances, and the amyloid status. Clinical diagnosis categories included cognitively normal (CN), mild cognitive impairment (MCI), or demented. A participant received a MCI diagnosis if their Mini Mental State Examination (MMSE) score was ≥ 24/30 accompanied by impaired performance in at least one cognitive domain (episodic memory, executive, visuo-spatial, and language). The cut-off criteria were set at −1.33 *Z*-score for memory and −2 *Z*-scores for other cognitive domains [13]. All demented individuals were characterized by an MMSE score <24, episodic memory impairment, and functional impairment in everyday life.

The amyloid status of participants was determined either by lumbar puncture (*n* = 55) or amyloid PET-scan with [^18^F]Flutemetamol (*n* = 33). In cerebrospinal fluid (CSF), measurements of amyloid Beta-42 (Aβ42), total tau (t-tau), and 181-phospho-tau (p-tau) proteins were conducted using Lumipulse automated assays. Amyloid PET-scans were quantified according to the centiloid method [14]. Subjects were considered to have AD pathology if at least one of these three conditions was met: flutemetamol PET Centiloid > 25 [14], CSF Aβ42 < 437pg/ml, [437 pg/ml < CSF Aβ42 <650pg/ml], and CSF P-tau >61 pg/ml [15]. Six participants had both CSF and amyloid-PET data available. Four of them had consistent, positive AD pathology results between modalities, and two had a positive amyloid PET but negative CSF (#1: Centiloid = 99.8; AB42 = 747pg/ml and P-tau = 141pg/ml; #2: Centiloid = 59.8; AB42 = 491pg/ml and P-tau = 59pg/ml). These two participants were considered as having amyloid pathology.

Subsequently, based on the combination of clinical and amyloid classifications, four bioclinical categories were defined as follows:

- CNamy − : amyloid-negative cognitively normal subjects.
- PreAD: preclinical AD, amyloid-positive cognitively normal subjects.
- ProAD: prodromal AD, amyloid-positive MCI subjects.
- ADdem: AD dementia, amyloid-positive demented subjects.

### PET and MRI acquisition

#### P-TAU PET/CT

[^18^F]MK-6240 (Cerveau Technologies, Knoxville TN) is an investigational drug studied as a second-generation cerebral tau tangles imaging agent.

Ninety minutes after intravenous administration of [^18^F]MK-6240 (target activity 185 ± 5 MBq) a 30-min dynamic list-mode acquisition was performed on a Philips Vereos digital PET-CT (Philips Healthcare, Amsterdam Netherlands). Images were reconstructed using manufacturer’s reconstruction algorithm which includes attenuation, scatter, and decay corrections, and time-of-flight information. Point spread function (PSF) and 1 mm re-slicing was also computed using the manufacturer’s algorithm to obtain a better resolution recovery [16].

#### Anatomical MRI sequence

Seventy-five of the 82 anatomical 3D T1-weighted MRI sequences were acquired on a 3-T MRI (GE Signa Premier 3T, GE Healthcare, Chicago IL) with a 48-channel phased-array head coil. Two different acquisition process were performed (acquisition #1 for 44 subjects and acquisition #2 for 31 subjects). For the seven remaining subjects, a previous anatomical 3D T1 MRI was available and has been used for the workflow analysis.

Acquisition #1: 196 slices were acquired using the following parameters: Repetition time (TR) = 7.2 ms, echo time (TE) = 2.9 ms, flip angle (FA) = 11°; slice thickness 1.2 mm; acquisition matrix 256 × 256, 196 slices; field of view (FOV) 270 × 270 mm^2^

Acquisition #2: 156 slices were acquired using the following parameters: TR = 2188.16 ms; TE = 2.96 ms; FA = 8°; slice thickness = 1 mm; acquisition matrix = 256 × 256, 156 slices; FOV = 256 × 256 mm^2^.

### PET image post-processing

Semi-quantitative analyses were performed using the PMOD 4.1 (PNEURO and PVIEW modules) software (PMOD LLC Technologies, Zurich Switzerland). The MRI was first segmented in 83 volumes of interest (VOIs) using the Hammers N30R83 Maximum Probability Atlas. Most peripheral voxels were masked using PMOD built-in CSF masking tool which was set to 0.3 (scale 0 to 1). Internal (white matter) voxels masking tool was left to default 0.5 value (scale 0 to 1). PET images were then matched on the segmented MRI and VOIs grouped following cumulative Braak stages resulting in three regions of interest: *Braak* ≤ *2*, *Braak* ≤ *4*, and *Braak* ≤ *6* based on previously defined regions-of-interest [17] ( Supplementary Material 1). All metrics were calculated in the native PET space. The SUV on the PET images were divided by the whole grey cerebellar SUV_mean_ to obtain SUV-ratio (SUVr) images. The mean SUVr value for each of the three Braak regions of interest was computed. Then, the extent of tauopathy (EOT) percentage was calculated by dividing the number of voxels with SUVr >1.3 by the total number of voxels in the same region. The cut-off value of 1.3 SUVr was selected using the 95th percentile of histogram values for a sample of ten visually negative tau PET images (Supplementary Material 2). This threshold best fitted the visual assessment of two trained nuclear physicians (TG, RL) and was confirmed in the literature as a positivity cut-off for other tau-PET radiopharmaceuticals [18]. Two alternative thresholds (SUVr = 1.2 and SUVr = 1.4) were also tested but did not show any improvement compared to the selected 1.3 cut-off. Notably, the semi-quantitative values obtained with the three different cut-offs exhibited very strong correlations (*R*^2^ > 0.988) **(**Supplementary Material 3).

**Fig. 1:**
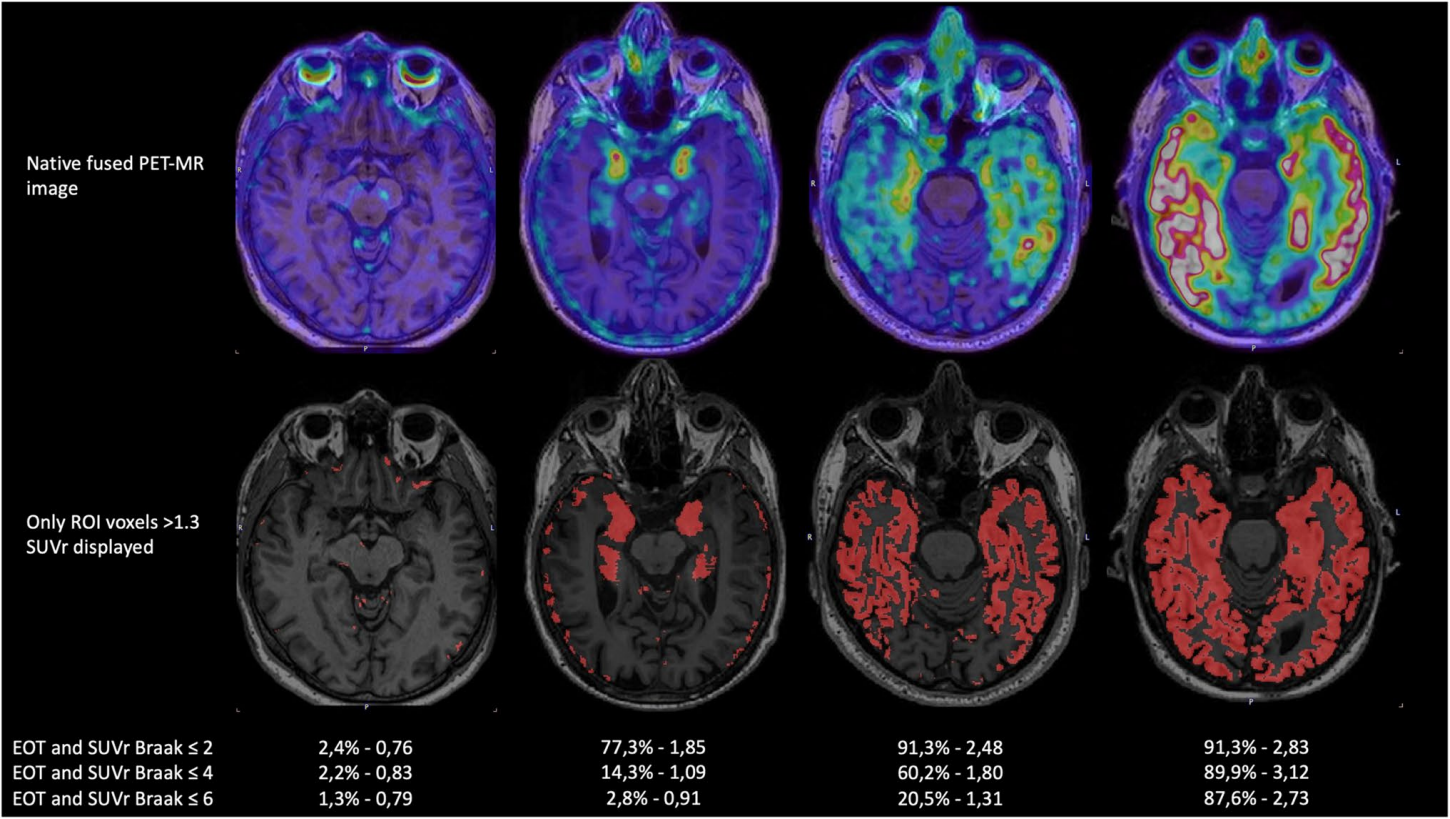
Illustration of typical stages of tauopathy spread. Illustration of four [^18^F]MK-6240 PET-scans matched to 3DT1 MRI. On the second line, the same scans are presented but only the voxels > 1.3 SUVr are displayed in monochromatic red, illustrating the extent of tauopathy (EOT) metric. The four scans illustrate typical Braak stages spread of AD (Braak 0, 2, 4, and 6). Semi-quantitative data are given for each region underneath the images

An illustration of PET-MR fused images displaying native PET and tauopathy extent (>1.3 SUVr ROI voxels) is shown on Figure 1 for four typical AD stages.

### Neuropsychological assessment

All participants underwent a comprehensive neuropsychological evaluation, and *Z*-scores were computed for four cognitive domains: episodic memory (Free and Cued Selective Reminding Test, French version [19]), language (Lexis Naming Test, the Category Fluency Test for animals, and the Letter Fluency Test for the letter ‘P’ [20]), executive functions (Trail Making Test, Luria’s Graphic Sequences (adaptations in French, unpublished)), and visuospatial functions (Clock Drawing Test and the Praxis part of the CERAD battery[21, 22]). Each cognitive domain was assessed based on three measures (for additional details about the neuropsychological assessment, refer to [13]). Subsequently, a global cognitive Z-score was calculated by averaging the *Z*-scores from these four cognitive domains. In this study, all analyses used the global cognitive *Z*-score as a proxy for cognitive performance/impairment.

### Statistics

All analyses were computed using the GraphPad Prism 9 software (Graphpad LLC, Boston MA) with two-tailed reported *p*-values. Data sets were tested for normality using Shapiro-Wilk test (data not shown). As a significant part of the data sets did not follow a normal distribution, we decided to provide non-parametric statistics for all analyses conducted in the study.

We first performed a direct Spearman’s correlation between EOT and SUVr values in each of the three Braak-stage regions. Then, we computed Kruskal-Wallis tests — with post hoc two-by-two Dunn’s multiple comparisons tests — to compare semi-quantitative results (SUVr and EOT) in each of the three Braak-stage regions. Post hoc tests were corrected for multiple comparisons. ROC curves were then processed to evaluate the diagnostic performance of [^18^F]MK-6240 tau-PET in the different bioclinical groups. ROC curves for four pairs of groups were plotted: CNamy− vs preAD (early pathological detection), CNamy− vs proAD (diagnostic detection), preAD vs proAD (early clinical detection), and proAD vs ADdem (dementia detection).

We then correlated the semi-quantitative results with the global cognitive *Z*-scores using simple linear regressions. We evaluated which of SUVr or EOT was most closely associated with cognitive performance in a multivariate model by using multiple regressions including both predictors. We conducted an analysis adjusted for age, sex, and ApoE4 carriage. The results remained unchanged after adjusting for these covariables (complete data not shown). Additionally, we performed a separate analysis adjusting for clinical stage. Finally, for the subset of subjects who had undergone lumbar puncture, the CSF t-tau/AB42 ratio was correlated with the global cognitive Z-score and the two PET metrics (EOT and SUVr) to compare how PET and CSF are respectively associated with cognition. We used the CSF t-tau/AB42 ratio, as it is a well-established AD biomarker [15] that was not used as an inclusion criterion (the CSF p-tau and AB42 concentrations were used for that purpose).

## Results

### Characteristics of the participants

The 82 subjects were classified following their categorical diagnosis into four bioclinical groups: CNamy− (*n* = 17), preAD (*n* = 14), proAD (*n* = 32), and ADdem (*n* = 19). The CNamy− group was significantly younger than the preAD and proAD groups (Table 1). All groups had a similar proportion of ApoE4 carriers and were not statistically different in terms of gender. CNamy− and preAD groups had significantly higher education levels than the proAD and ADdem groups (16.0 and 15.7 years vs 13.6 and 14.3 years, respectively).

When comparing groups based on cognitive data, CNamy− and preAD had non-significantly different MMSE but had distinct cognitive *Z*-scores (+0.31 vs -0,15, *p* = 0.003). This is consistent with the notion that the global cognitive *Z*-score, resulting from an average of the neuropsychological battery, provides a finer analysis of cognitive performances than the MMSE, especially for subtle deficits among CN individuals. As per definition, participants in the proAD and ADdem groups displayed worse cognition than CNamy−.

Comparing amyloid biomarkers data, all groups were, by definition, different from CNamy− in terms of flutemetamol-PET centiloid values. Although not reaching statistical significance, centiloid values were higher in ADdem than in proAD (*p* = 0.07) and higher in proAD than in preAD (*p* = 0.08), while CSF amyloid values were similar in pre-AD, proAD, and ADdem. The CSF total tau and p-tau values did not show significant differences, with ADdem not being significantly higher than in proAD (total tau: *p* = 0.20, p-tau: *p* = 0.10) and proAD not being significantly higher than in preAD (total tau: *p* = 0.19, p-tau *p* = 0.19).

### [^18^F]MK-6240 PET metrics

The first [^18^F]MK-6240 PET inter-group analysis compared grey cerebellum SUV in the different groups and demonstrated no statistical difference in terms of cerebellar SUV across the different groups (Table 1). As this metric is used as the reference region for SUVr calculation and subsequently for all analyses, it was crucial to demonstrate a consistency of the cerebellar reference regions among groups. This region is supposed to be not affected by tau pathology, and thus, cerebellar SUVmean is traditionally employed as the reference region for SUVr computation.

**Fig. 2:**
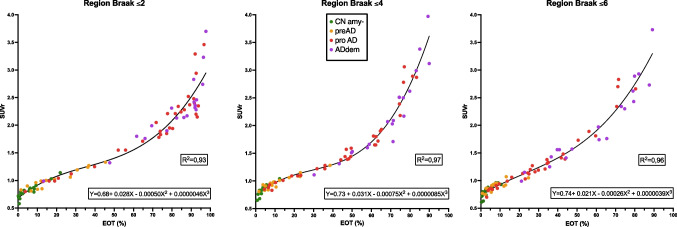
Correlation between EOT and SUVr. Correlation graphs between extent of tauopathy (EOT) and SUVr metrics in the three regions of interest. Equations and *R*^2^ are given for the third-degree polynomial fit. CNamy − , cognitively normal amyloid-negative subjects; preAD, cognitively normal amyloid-positive subjects; proAD, mild cognitively impaired amyloid-positive subjects; ADdem, amyloid-positive demented subjects

The EOT and SUVr metrics were correlated to each other. The two quantification indexes were, as expected, quite strongly correlated with linear Pearson’s *R*^2^ of 0.89, 0.85, and 0.92 in *Braak* ≤ *2*, *Braak* ≤ *4*, and *Braak* ≤ *6* regions, respectively*.* Using a non-parametric test (Spearman’s correlation) even higher *R*^2^ were obtained at 0.95, 0.97, and 0.98, respectively. The correlation tended to follow a third-degree polynomial curve with EOT increasing before SUVr (Figure 2).

### Group analysis of tau PET quantification (Figure 3; Table 2)

**Fig. 3:**
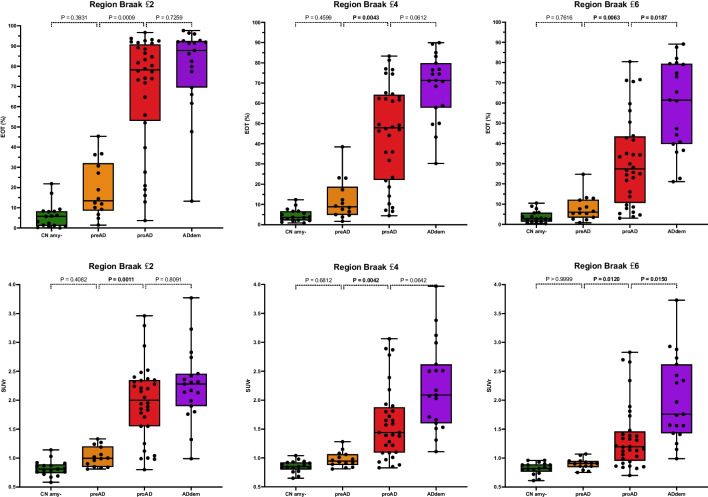
Percentage of tauopathy and SUVr for the three clusters of regions. Bioclinical group comparison of extent of tauopathy (EO)T and SUVr metrics in the three Braak regions of interest. Kruskal–Wallis test was performed to compare groups (*p* < 0.0001 for all) and Dunn’s multiple comparison test to compare each bioclinical group with the following one. Two-tailed *p*-values are displayed on the graph. CNamy − , cognitively normal amyloid-negative subjects; preAD, cognitively normal amyloid-positive subjects; proAD, mild cognitively impaired amyloid-positive subjects; ADdem, amyloid-positive demented subjects

We observed a clear and gradual scaling of tau-PET signal between clinical stages (either quantified by EOT or SUVr). To illustrate, mean EOT in *Braak* ≤ *6* region is 3.7% for CNamy−; 7.9% for preAD, 31.5% for proAD, and 59.0% for ADdem. The Kruskal-Wallis test revealed significant differences between groups in all regions for both EOT and SUVr (*p* < 0.0001). Post hoc paired comparisons demonstrated significant differences between the preAD and proAD groups for both EOT and SUVr, in all regions-of-interest. Additionally, we observed significant differences between the proAD and ADdem groups in the *Braak* ≤ *6* region. Although the difference between CNamy− and preAD was most pronounced in the *Braak* ≤ *2* region (mean 6.0% vs 19.0%), it did not reach statistical significance (*p* = 0.39) using non-parametric statistics (Figure 3).

**Table 2:** Tau PET metrics mean ± SD for each bioclinical group

		Region Braak ≤ 2	Region Braak ≤ 4	Region Braak ≤ 6
EOT (%)	CNamy −	6.0 ± 6.1	4.6 ± 3.1	3.8 ± 3.0
	preAD	19.0 ± 13.8	12.3 ± 10.1	7.0 ± 6.2
	proAD	68.0 ± 28.3	45.6 ± 24.8	31.5 ± 22.3
	ADdem	79.7 ± 21.0	68.5 ± 16.2	59.0 ± 22.2
SUVr	CNamy-	0.82 ± 0.13	0.86 ± 0.10	0.82 ± 0.10
	preAD	1.02 ± 0.18	0.98 ± 0.13	0.90 ± 0.09
	proAD	1.96 ± 0.96	1.60 ± 0.63	1.36 ± 0.57
	ADdem	2.26 ± 0.63	2.23 ± 0.76	2.02 ± 0.73

Extent of tauopathy (EOT) and SUVr mean ± SD values for each bioclinical group in each of the three Braak regions

*CNamy − *cognitively normal amyloid-negative subjects, *preAD* cognitively normal amyloid-positive subjects, *proAD* mild cognitively impaired amyloid-positive subjects, *ADdem* amyloid-positive demented subjects

### ROC curves (Figure 4; Suppl. 4)

**Fig. 4:**
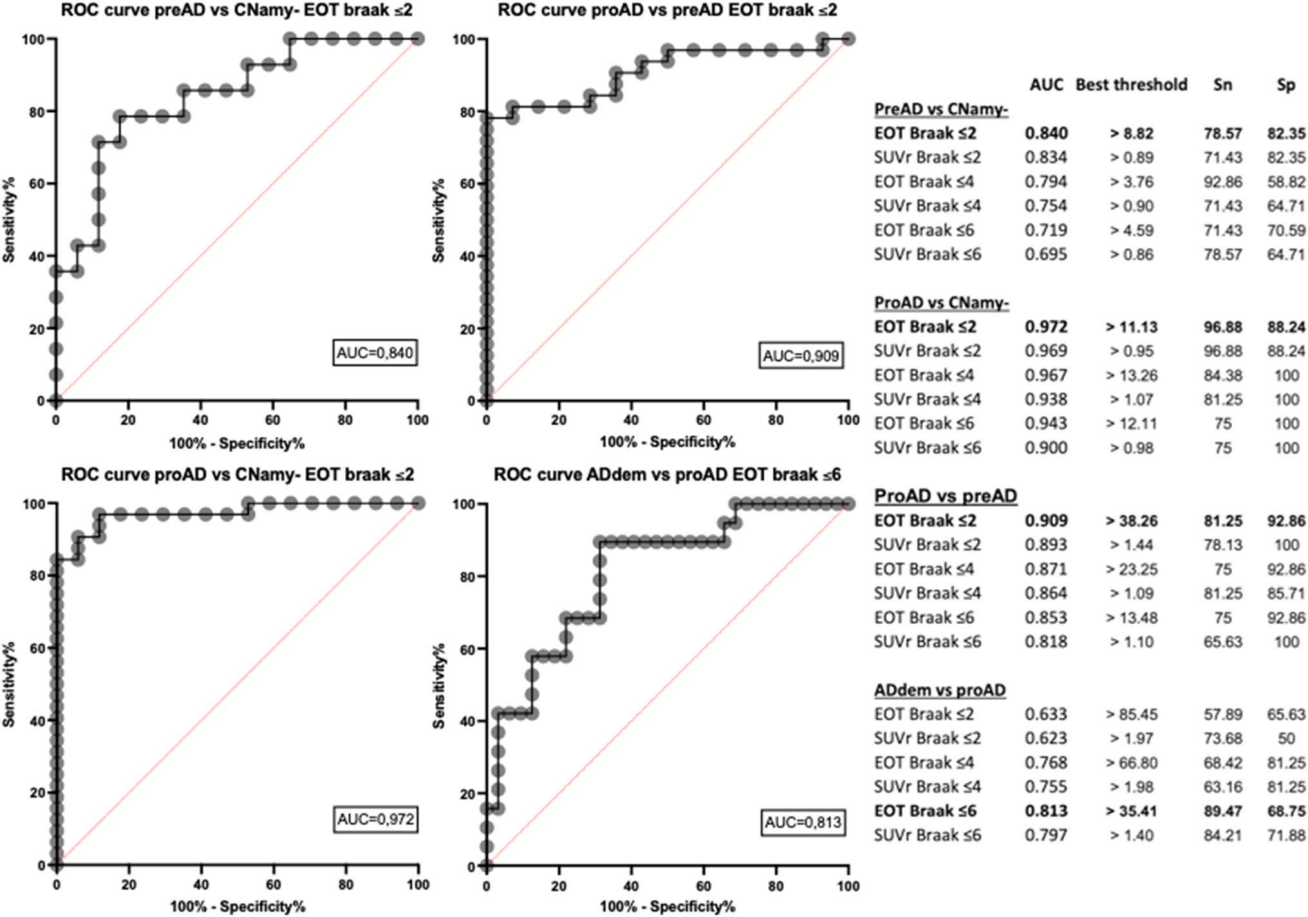
ROC curves. ROC curve data table evaluating the two [^18^F]MK-6240 PET metrics (extent of tauopathy (EOT) and SUVr) to distinguish subjects of the four bioclinical groups. The best AUC curve for each Braak region is displayed on graph. CNamy − , cognitively normal amyloid-negative subjects; preAD, cognitively normal amyloid-positive subjects; proAD, mild cognitively impaired amyloid-positive subjects; ADdem, amyloid-positive demented subjects

For all ROC curves, areas under the curve (AUC) for EOT and SUVr were similar, with consistently higher values for EOT (∆_AUC_: +0.003 to +0.043). Delong test revealed that the differences in AUC between EOT and SUVr were not statistically significant (0.06 ≤ *Z* ≤ 0.72). When distinguishing between CNamy− and preAD, CNamy− and proAD, and preAD and proAD, the optimal PET marker was EOT in *Braak* ≤ *2* region (AUC respectively 0.84; 0.97 and 0.91). In this region, a threshold of 8.8% EOT was more effective in distinguishing between CNamy− and preAD, while a threshold of 38.3% was optimal for distinguishing between preAD and proAD (see Figure 4 for sensitivity and specificity at those thresholds and a thorough comparison of all PET biomarkers analyzed). To discriminate between proAD and Addem, the most effective PET biomarker was EOT in the *Braak* ≤ *6* region (AUC = 0.81) with a threshold of 35.4% for tau-PET signal positivity across the entire cortex.

The highest accuracy was observed when comparing CNamy− and proAD using EOT in the *Braak* ≤ *2* region (AUC = 0.97 with a threshold of 11.1%). It is worth noting that we also calculated ROC curves for CNamy− versus the subjects within the AD spectrum (preAD + proAD + ADdem) and versus impaired AD subjects (proAD + ADdem). The results were comparable to those presented here (Supplementary material 4).

### Correlation between tau-PET quantification and cognitive performances(Figure 5; Table 3)

**Fig. 5:**
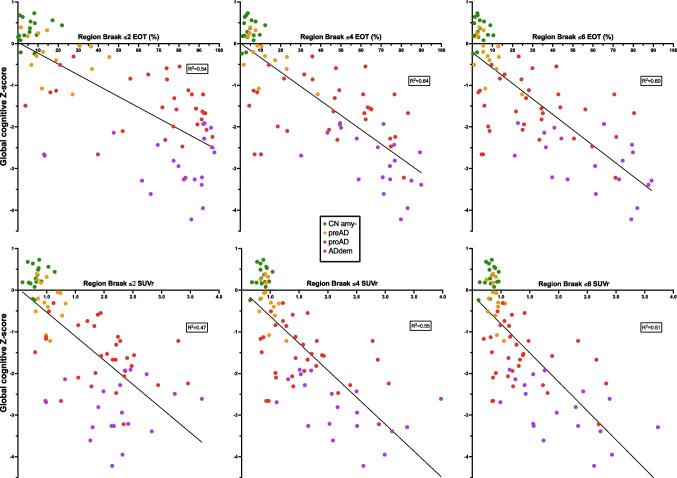
EOT and SUVr simple linear regression with global cognitive *Z*-scores. Simple linear regressions of PET metrics (extent of tauopathy (EOT) and SUVr) and global cognitive *Z*-scores in the three Braak regions of interest. *R*^2^ are given on each graph. CNamy − , cognitively normal amyloid-negative subjects; preAD, cognitively normal amyloid-positive subjects; proAD, mild cognitively impaired amyloid-positive subjects; ADdem, amyloid-positive demented subjects

**Table 3:** Multivariate analysis

Parameter estimates	Variable	Estimate	Standard error	95% CI (asymptotic)	|*t*|	*p* value
Region Braak ≤ 2						
* β*0	Intercept	− 0.06796	0.3029	− 0.6708 to 0.5349	0.2244	0.823
* β*1	EOT Braak ≤ 2	− 0.02903	0.008221	− 0.04540 to -0.01267	3.532	0.0007
* β2*	SUVr Braak ≤ 2	0.1511	0.3915	− 0.6282 to 0.9303	0.3859	0.7006
Region Braak ≤ 4						
* β*0	Intercept	0.01119	0.2449	− 0.4762 to 0.4986	0.04571	0.9637
* β*1	EOT Braak ≤ 4	− 0.03456	0.007687	− 0.04986 to -0.01926	4.496	< 0.0001
* β*2	SUVr Braak ≤ 4	− 0.0007859	0.3114	− 0.6206 to 0.6190	0.002524	0.998
Region Braak ≤ 6						
* β*0	Intercept	− 0.627	0.3401	− 1.304 to 0.04994	1.844	0.069
* β*1	EOT Braak ≤ 6	− 0.05201	0.01176	− 0.07542 to -0.02859	4.421	< 0.0001
* β*2	SUVr Braak ≤ 6	0.6283	0.4768	− 0.3209 to 1.577	1.318	0.1914

Multivariate analysis of extent of tauopathy (EOT) and SUVr to predict global cognitive *Z*-scores in the three regions of interest *Braak* ≤ *2, Braak* ≤ *4,* and* Braak* ≤ *6*

We observed strong correlations between EOT and the global cognitive *Z*-score with *R*^2^ of respectively 0.55, 0.64, and 0.60 for the *Braak* ≤ *2*, *Braak* ≤ *4*, and *Braak* ≤ *6* regions. Those correlations consistently outperformed the results obtained with the SUVr metric (*R*^2^ values of 0.47, 0.55, and 0.51 in the *Braak* ≤ *2*, *Braak* ≤ *4*, and *Braak* ≤ *6* regions, respectively). Consequently, the EOT in the *Braak* ≤*4* region provided the most comprehensive explanation of cognitive tests results, accounting for 64% of the variance. Furthermore, in a multivariate analysis where both EOT and SUVr were simultaneously entered as predictors of cognitive performance (Table 3), EOT consistently demonstrated an association with cognition for every region, even after adjusting for SUVr values. This underscores a closer relationship between EOT and cognition compared to the association between SUVr and cognition. When adjusting for clinical stage, the association between cognitive performances and EOT in the *Braak* ≤ *2* region is no longer statistically significant (*R*^2^ = 0.22). However, the association remains significant for the *Braak* ≤ *4* and *Braak* ≤ *6* regions (*R*^2^ = 0.0022 and 0.00033, respectively). We also investigated the effect of ApoE carriage in the subgroup of subjects with known status, focusing on *Braak* ≤ *6* EOT. The correlation was notably stronger in the ApoE4+ group (*R*^2^ = 0.56) compared to the ApoE4− group (*R*^2^ = 0.18). However, the ApoE+ group included 32 subjects, with 15 showing impairment, while the ApoE- group comprised 18 subjects, with 7 exhibiting impairment.

It is worth noting that we also tested a pure intensity index using the mean SUVr of the voxels >1.3 (SUVr_mean > 1.3_) in each region and a “total lesion glycolysis-like” index (EOT × SUVr_mean > 1.3_). However, the correlation with cognitive *Z*-scores was found to be inferior (*R*^2^ of 0.41, 0.51, and 0.46 for SUVr_mean > 1.3_ and 0.49, 0.57, and 0.47 for (EOT × SUVr_mean > 1.3_) in *Braak* ≤ *2*, *Braak* ≤*4*, and *Braak* ≤ *6* regions, respectively. Complete data not shown).

Regarding the choice of cumulative regions, we assessed the correlation of EOT Braak ≤ 4 with EOT Braak 3–4 (excluding Braak 1–2 regions) and the correlation was found to be excellent (*R*^2^ = 0.997, *p* < 0.0001). Similarly, the correlation of EOT Braak ≤ 6 with EOT Braak 5–6 (excluding Braak 1–2–3–4 regions) was very good (*R*^2^ = 0.970, *p*<0.0001). However, from a clinical practice perspective, using a global aggregate (Braak ≤ 6) requires less processing than using a Braak 5–6 aggregate, making it potentially easier to use in clinical practice.

### CSF (Figure 6)

**Fig. 6:**
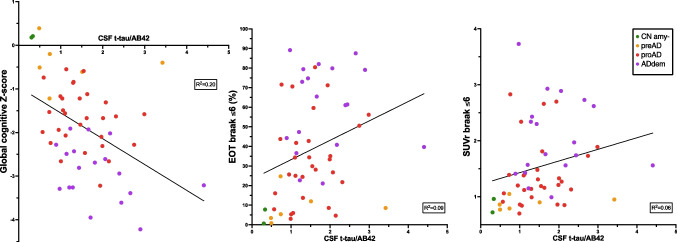
Correlation between CSF t-tau/AB42 ratio, cognitive performances, and PET metrics. Simple linear regressions of CSF t-tau/AB42 ratio and global cognitive *Z*-scores in the three Braak regions of interest. *R*^2^ are given on each graph. CNamy − , cognitively normal amyloid-negative subjects; preAD, cognitively normal amyloid-positive subjects; proAD, mild cognitively impaired amyloid-positive subjects; ADdem, amyloid-positive demented subjects

The correlation between CSF t-tau/AB42 and cognitive performances was analyzed for the subgroup of participants with CSF data available (*n* = 55). The correlation was significantly lower than what was observed for [^18^F]MK-6240 with an *R*^2^ of only 0.20 between CSF and cognition. The correlations between t-tau/AB42 and PET metrics (EOT and SUVr) were also weak, with respective *R*^2^ values of 0.09 and 0.06. It is noteworthy that using CSF t-tau or p-tau instead of the t-tau/AB42 ratio did not improve the correlation with cognition (*R*^2^ of 0.17 and 0.19, respectively; data not shown).

## Discussion

This study aimed to investigate the correlation between [^18^F]MK-6240 tau PET and cognitive performances as well as to assess the potential added-value of a new PET metric, the extent of tauopathy. This metric was designed to better reflect the regional extension of tau pathology revealed by [^18^F]MK-6240 PET than the usual SUVr semi-quantification metric. This study was conducted within a preselected AD spectrum population.

### PET semi-quantitative results and categorial diagnosis

Our results showed a clear progression of the measured tauopathy (either by EOT or SUVr) with increasing bioclinical stages of AD. Furthermore, these measures, and particularly EOT, proved to be effective to differentiate MCI patients from clinically normal subjects. According to ROC curves, the best region to analyze in order to diagnose the early AD stages (preAD or proAD) was *Braak* ≤ 2. Within the cognitively normal population, a cut-off of 8.8% EOT in *Braak* ≤ *2* could indeed separate the group into amyloid-positive and amyloid-negative with a sensitivity of 79% and a specificity of 82%. Based on this analysis, tau-PET could partially replace amyloid-PET using a low threshold for positivity in the mesiotemporal lobe. Indeed, amyloid-negative CN individuals very rarely exhibited significant positivity on [^18^F]MK-6240 tau-PET.

Within the amyloid-positive population (CN and MCI), using a *Braak* ≤ *2* EOT cut-off of 38% enabled to distinguish CN from MCI subjects with a sensitivity of 81% and a specificity of 93%. It is particularly interesting to note that if an amyloid-positive patient presents a clinically relevant cognitive impairment (MCI), [^18^F]MK-6240 PET will, in almost every case, show a certain degree of tauopathy in the mesiotemporal lobe. On the other hand, CNamy− subjects will not show significant *Braak* ≤ *2* tau PET signal. Our results suggest that unlike the widely used Flortaucipir ([^18^F]AV-1451) [12], [^18^F]MK-6240 seems to be a reliable diagnostic biomarker for AD-related mesiotemporal tauopathy and therefore in early AD stages. Importantly, a higher threshold was used to detect cognitively impaired patients, highlighting that the threshold selection depends on the purpose of the analysis.

### PET semi-quantitative results and cognitive performances

Tau PET showed a strong correlation with global cognitive *Z*-scores. More than half of the variance in cognitive results (up to 64%) was explained by tau PET semi-quantitative measures. Notably, in this situation, the EOT metric systematically outperformed SUVr (Δ*R*^2^ ranged from +0.08 to +0.09). This suggests that the extent of tauopathy was better associated with cognitive performances than the average intensity of the [^18^F]MK-6240 uptake in cortical voxels. This is corroborated by the inferior correlations obtained with the pure intensity index (SUVr_mean > 1.3_) or the “total lesion glycolysis-like” index (EOT × SUVr_mean > 1.3_). The pure intensity metric showed the worst results when correlated with cognitive performances and the addition of the intensity to EOT worsened the correlation.

The metric most strongly associated with cognitive performances in our study was the EOT in the *Braak* ≤ *4* region. This region approaches the definition of the previously validated meta-ROI [23]. The superiority of the *Braak* ≤ *4* region in our study likely stems, at least in part, from our predominantly CN and MCI population, rather than demented subjects. Indeed, MCI (and CN individuals, naturally) are not expected to show extensive — if any — tauopathy in Braak 5 or 6 regions. Consequently, including these large regions in the quantification may not yield additional information to explain the cognitive performances of these patients. Nevertheless, the EOT in the *Braak* ≤ *6* region was the second-best metric associated with cognitive performances. The EOT measure in this global region has the advantage of not involving any assumption on region delineation as it quantifies the tauopathy on the whole cortex (plus accessory regions, some of the grey nuclei; see Supplementary Material 2). On the other hand, semi-quantitative metrics in *Braak* ≤ *2* region is the most useful for diagnostic purposes but, as the region is small, it quickly saturates and blinds us to further progression of the disease and concurrent cognitive decline. Moreover, due to the size of the region, differences in performances between PET metrics SUVr and EOT quantification tend to fade.

When compared to SUVr (Figure 2), EOT allowed finer discrimination between subjects in the 15 to 60% range of extent — especially for the *Braak* ≤ *4* region. Within the narrow SUVr range of (1.05 to 1.65, coefficient of variation = 1.57) for this region (while the complete range of SUVr values in our study is [0.65 to 3.97]), EOT exhibited a broader variation, ranging from 15 to 60% (coefficient of variation = 4.00). This range is precisely the range of clinical interest for MCI patients. Indeed, in the *Braak* ≤ *4* region, under a threshold of 15% EOT, most subjects were cognitively unimpaired, and above 60%, most subjects were either demented or had serious cognitive impairment with low cognitive *Z*-scores (at least −2 *Z*-scores). The main clinical interest of [^18^F]MK-6240 PET is therefore for patients presenting mild or subjective cognitive impairment. For those patients, it is very clinically relevant to first confirm or reject AD diagnosis, and then to determine the extent (or progression) of cerebral tauopathy. Therefore, not only does EOT correlated better with cognition than SUVr in *Braak* ≤ *4* region, but it also gave a more precise measure of the cerebral tauopathy in the 15% to 60% range. Regarding the *Braak* ≤ *2* region, due to its small size, the results were mostly dichotomous, and most of the subjects had an EOT of either <20% or >60%. This may explain why the correlation results are no longer significant in this region when adjusted for clinical stage.

### Observed tauopathy, neuronal death, and cognitive decline

Based on our current knowledge of AD physiopathology, it is reasonable to assume that a temporal gap likely exists between the development of cortical tauopathy and the neuronal death leading to cognitive impairment. This could explain why some subjects with extended tauopathy (i.e., more than 40% *Braak* ≤ *6* EOT) still have decent cognitive performances (less than −2 *Z*-scores). The underlying biological reality behind the observed tauopathy likely involves a mix of regions with older tau tangles accumulation and subsequent neuronal death, alongside regions with newer tau tangles accumulation where a certain level of neuronal function is preserved. As we conducted a cross-sectional study, we were unable to discern this phenomenon, that ongoing longitudinal studies are investigating. In the future, a direct voxel-based comparison between [^18^F]Fluoro-deoxy-glucose ([^18^F]FDG) and [^18^F]MK-6240 PET biodistribution may help to explore such discrepancies.

### Tau PET semi-quantitative measures versus visual reading

While some may argue that PET visual reading provides sufficient information for clinicians and may spare a 3DT1 MRI, we believe that semi-quantitative measures provide an important added value. Firstly, they provide a reader-independent continuous value, in opposition to visual reading, which is a discrete variable (either Braak stage or other staging method [24]). This continuous information is more detailed and facilitates precise longitudinal comparisons, whether in pharmacological trials or natural history evolution contexts. Moreover, recent studies [25] have demonstrated different tau deposition trajectories. Using semi-quantification in the *Braak* ≤ *6* region preserves the measure from any physiopathological assumption on the temporality and pattern of tau spread. Indeed, using a global measure such as *Braak* ≤ *6* EOT quantifies every significative voxel of tau deposit independently of its location and individual distribution pattern. In contrast, visual reading classification may be challenging for some distribution patterns (i.e., mesiotemporal sparing). Additionally, this global measure is easily reproductible and may help to better compare patients enrolled in different clinical trials. However, it is important to acknowledge that further investigations are necessary to understand the influence of the software workflow on the EOT quantification.

### Study limitations and outlooks

Firstly, our study population, especially the volunteers, had a higher socio-economic status compared to the average population. This is mainly due to the geographical location of the hospital and the method of recruitment. Notably, CNamy− subjects had slightly superior cognitive performances than our historical independent control group [13] with an average *Z*-score at +0.31. Additionally, they were younger than the patients (with a mean age difference of −9 years) making them less prone to tauopathy, given that it increases with age. This is a result of classifying CN volunteers into CNamy− and preAD, as amyloid pathology increases with age. To partially overcome this limitation, we computed comparisons between AD patients and preAD, whose ages were similar.

Regarding the quantification method, we opted for an arbitrary 1.3 SUVr cut-off to determine “significant” voxels above background physiological uptake, noise, non-specific binding, and spill-over effect. The latter was the most challenging effect to manage. The meningeal and osseous [^18^F]MK-6240 binding may indeed be highly intense in some subjects (while almost absent in others), leading to a significative spill-over effect on the adjacent cortex. This challenge was particularly pronounced in the mesiotemporal region due to its anatomical boundaries. Consequently, a CNamy− subject, despite lacking a visually specific cortical signal, could exhibit up to 22% EOT in the *Braak* ≤ *2* region due to this effect, even with implemented strategies to mitigate it (PSF computing, the 1.3 filter, and the removal of the most peripheral voxels from the VOIs). We experimented different cut-offs (1.2; 1.4) for comparison, but the results did not show superior correlation with cognitive performances (data not shown).

Lastly, our study only included AD-spectrum subjects, rendering it unable to address the potential binding of [^18^F]MK-6240 to other tauopathies. Consequently, our findings do not provide insights into the diagnostic performance of [^18^F]MK-6240 tau PET in populations with unknown amyloid status or in clinically impaired patients with non-AD tauopathies.

The main outlooks for future research include longitudinal analyses of PET metrics and cognitive scores evolutions to better understand the role of tau spread in cognitive decline due to AD. Additionally, exploring a *Z*-map-based approach for tauopathy quantification holds promise, although this would require a larger control group with subjects spanning different ages. Lastly, testing the spatial extent approach on non-AD tauopathies would be particularly interesting, allowing to determine the specificity of [^18^F]MK-6240 semi-quantification for AD diagnosis.

## Conclusion

The semi-quantitative results from [^18^F]MK-6240 tau PET show strong association with cognitive performances within an AD-spectrum population. The computation of a pure extent metric, such as EOT, significantly improves the correlation compared to the commonly used SUVr metric, which combines information about both extent and intensity. Mapping the extent of cerebral tauopathy could thus be a novel and improved approach for tau PET quantification in a neurocognitive context.

Furthermore, our study highlights the efficacy of [^18^F]MK-6240 tau-PET semi-quantification in the mesiotemporal region (*Braak* ≤ *2*) for bioclinical diagnostic purposes. Specifically, it accurately distinguishes between amyloid-positive and amyloid-negative subjects in the CN population, and between MCI and CN subjects in the amyloid positive population.

### Supplementary Information

Below is the link to the electronic supplementary material.

Supplementary file1 (DOCX 1525 KB)

Supplementary file2 (XLSX 27 KB)

## Data Availability

All data used in this article are available in an additional file.
