# Supplementary material for: The spatial extent of tauopathy on [18F]MK-6240 tau PET shows stronger association with cognitive performances than the standard uptake value ratio in Alzheimer’s disease
Source: Eur J Nucl Med Mol Imaging. 2024 Jan 17;51(6):1662–74. doi: 10.1007/s00259-024-06603-2 (PMC11043108; doi:10.1007/s00259-024-06603-2)

**Supplementary Material**

Supplementary Material 1: List of bilateral Hammers N30R83 VOIs composing each region in PMOD 4.1 and abbreviations correspondence.


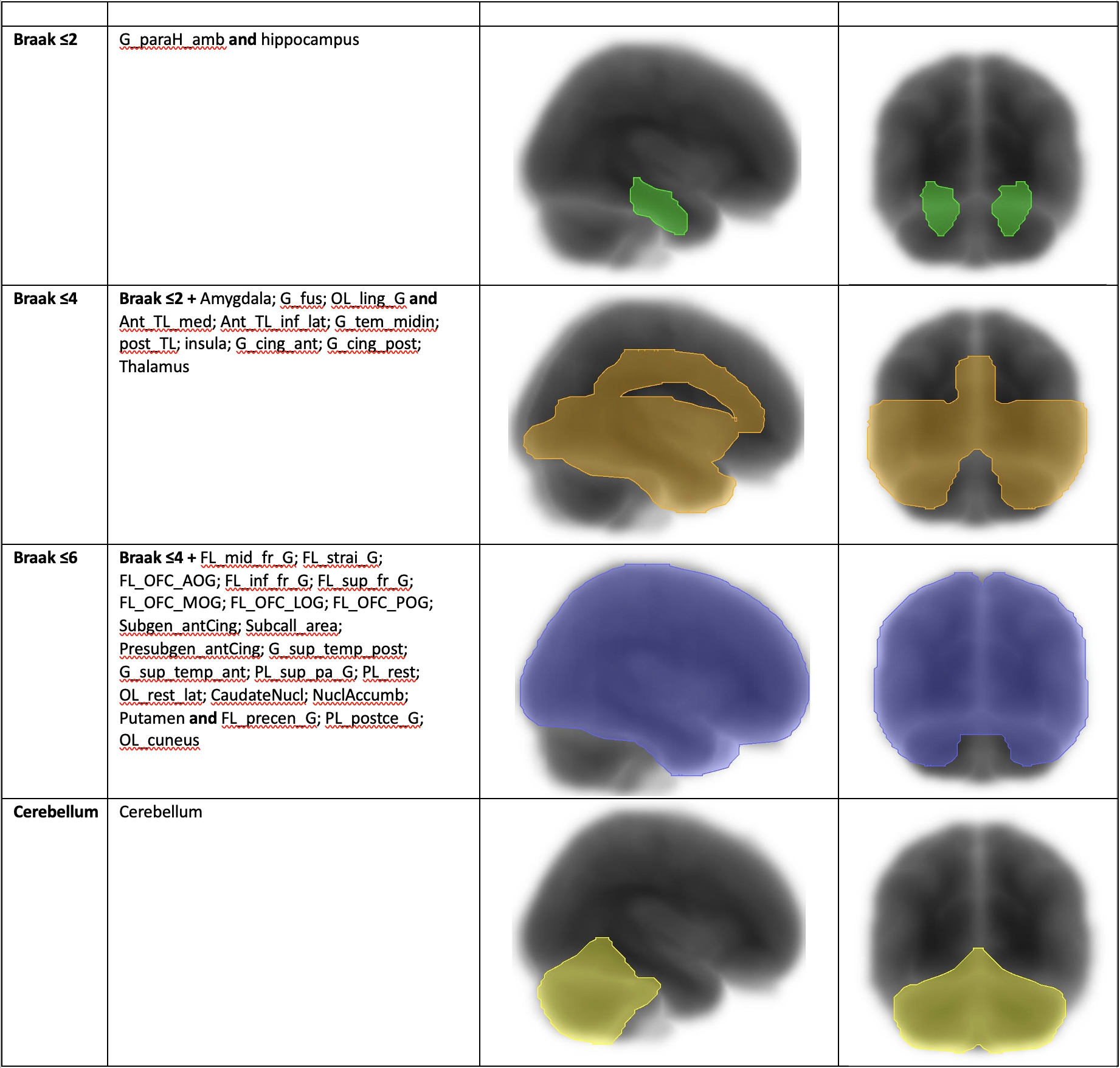


| **Abbreviation** | **Name** |
| --- | --- |
| Cerebellum | Cerebellum |
| Brainstem | Brainstem |
| Hippocampus | Hippocampus |
| Amygdala | Amygdala |
| CaudateNucl | Caudate nucleus |
| NuclAccumb | Nucleus accumbens |
| Putamen | Putamen |
| Thalamus | Thalamus |
| Pallidum | Pallidum |
| Corp_Callosum | Corpus callosum |
| S_nigra | Substantia nigra |
| FL_mid_fr_G | Middle frontal gyrus |
| FL_precen_G | Precentral gyrus |
| FL_strai_G | Straight gyrus |
| FL_OFC_AOG | Anterior orbital gyrus |
| FL_inf_fr_G | Inferior frontal gyrus |
| FL_sup_fr_G | Superior frontal gyrus |
| FL_OFC_MOG | Medial orbital gyrus |
| FL_OFC_LOG | Lateral orbital gyrus |
| FL_OFC_POG | Posterior orbital gyrus |
| Subgen_antCing | Subgenual frontal cortex |
| Subcall_area | Subcallosal area |
| Presubgen_antCing | Pre-subgenual frontal cortex |
| Ant_TL_med | Anterior temporal lobe medial part |
| Ant_TL_inf_lat | Anterior temporal lobe lateral part |
| G_paraH_amb | Parahippocampal and ambient gyri |
| G_sup_temp_post | Superior temporal gyrus posterior part |
| G_tem_midin | Middle and inferior temporal gyrus |
| G_fus | Fusiform gyrus |
| Post_TL | Posterior temporal lobe |
| G_sup_temp_ant | Superior temporal gyrus anterior part |
| PL_postce_G | Postcentral gyrus |
| PL_sup_pa_G | Superior parietal gyrus |
| PL_rest | Inferiolateral remainder of parietal lobe |
| OL_rest_lat | Lateral remainder of occipital lobe |
| OL_ling_G | Lingual gyrus |
| OL_cuneus | Cuneus |
| Insula | Insula |
| G_cing_ant | Cingulate gyrus anterior part |
| G_cing_post | Gyrus cinguli posterior part |

Supplementary Material 2: Histograms for 10 visually negative participants

Supplementary Material 3: Correlation matrix of EOT values for the three different regions and cut-offs, Spearmann r values are displayed.

Supplementary Material 4: ROC Curves results of CNamy- vs AD spectrum and AD impaired groups (similar to figure 4).


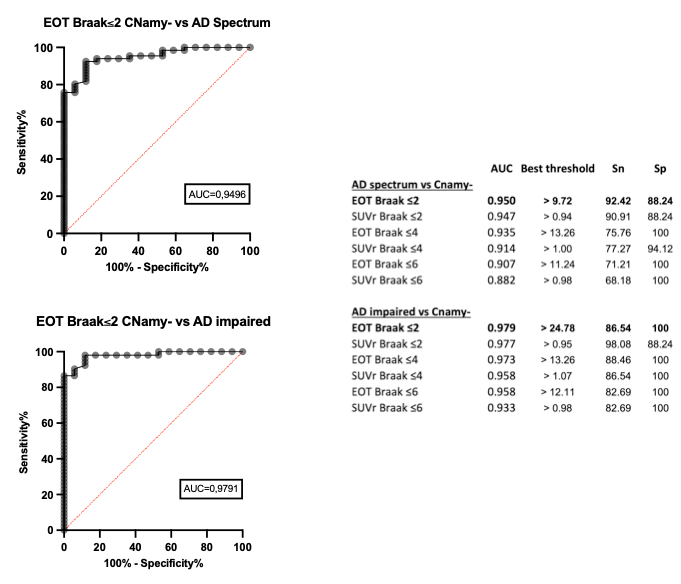

Supplement: Supplementary file 1 — Supplementary file1 (DOCX 1525 KB) [file 259_2024_6603_MOESM1_ESM.docx]
